# Association of HLA-B Gene Polymorphisms with Type 2 Diabetes in Pashtun Ethnic Population of Khyber Pakhtunkhwa, Pakistan

**DOI:** 10.1155/2021/6669731

**Published:** 2021-06-16

**Authors:** Asif Jan, Muhammad Saeed, Muhammad Hussain Afridi, Fazli Khuda, Muhammad Shabbir, Hamayun Khan, Sajid Ali, Muhammad Hassan, Samiullah, Rani Akbar, Zakiullah

**Affiliations:** ^1^Department of Pharmacy, University of Peshawar, Pakistan; ^2^Diabetes and Endocrinology Unit, Hayatabad Medical Complex Peshawar, Pakistan; ^3^Internal Medicine, College of Medicine, Shaqra University, Saudi Arabia; ^4^Department of Biotechnology, Abdul Wali Khan University, Mardan, Pakistan; ^5^Pakistan Air Force Hospital, Fort Munro, Pakistan; ^6^Department of Pharmacy, Abdul Wali Khan University, Mardan, Pakistan

## Abstract

Human leukocyte antigen (HLA) system is the most polymorphic and gene dense region of human DNA that has shown many disease associations. It has been further divided into HLA classes I, II, and III. Polymorphism in HLA class II genes has been reported to play an important role in the pathogenesis of type 1 diabetes (T1D). It also showed association with T2D in different ethnic populations. However, a little is known about the relationship of HLA class I gene polymorphism and T2D. This study has evaluated the association of HLA-B (class I gene) variants with T2D in Pashtun ethnic population of Khyber Pakhtunkhwa. In the first phase of the study, whole exome sequencing (WES) of 2 pooled DNA samples was carried out, and DNA pools used were constructed from 100 diabetic cases and 100 control subjects. WES results identified a total of *n* = 17 SNPs in HLA-B gene. In the next phase, first 5 out of *n* = 17 reported SNPs were genotyped using MassARRAY® system in order to validate WES results and to confirm association of selected SNPs with T2D. Minor allele frequencies (MAFs) and selected SNPs×T2D association were determined using chi-square test and logistic regression analysis. The frequency of minor C allele was significantly higher in the T2D group as compared to control group (45.0% vs. 13.0%) (*p* = 0.006) for rs2308655 in HLA-B gene. No significant difference in MAF distribution between cases and controls was observed for rs1051488, rs1131500, rs1050341, and rs1131285 (*p* > 0.05). Binary logistic regression analyses showed significant results for SNP rs2308655 (OR = 2.233, CI (95%) = 1.223‐4.077, and *p* = 0.009), while no considerable association was observed for the other 4 SNPs. However, when adjusted for these variants, the association of rs2308655 further strengthened significantly (adjusted OR = 7.485, CI (95%) = 2.353‐23.812, and *p* = 0.001), except for rs1131500, which has no additive effect. In conclusion, the finding of this study suggests rs2308655 variant in HLA-B gene as risk variant for T2D susceptibility in Pashtun population.

## 1. Introduction

Type 2 diabetes (T2D) is a multifactorial metabolic disease characterised by impaired glucose haemostasis that is primarily caused by lack of response of peripheral tissues to insulin and/or insufficient production/secretion of insulin by *β* cells of pancreas. Environmental and genetic variations are key risk factors for T2D [1, 2]. According to the recent report (Diabetes Atlas edition 2019) of International Diabetes Federation (IDF), approximately 415 million people around the world have diabetes, with 90% of these individuals having T2D [3]. Currently, the epicentres of diabetes mellitus (DM) prevalence have been in China, India, United States of America (USA), Pakistan, and Brazil [4]. Its prevalence rate is alarmingly high in developing countries, with more than 80% cases being reported in these nations [5]. It is projected that by 2045, the number of cases in Pakistan will exceed that of in the USA, moving the former from 4^th^ position to 3^rd^ in diabetes prevalence race [3]. Currently in Pakistan, 19.4 million people are living with diabetes [3]; the number was 5.5 million in 2000 [6].

Type 2 diabetes (T2D) risk is strongly heritable [7–9]. Genetic studies offer a powerful approach for better screening and treatment of diseases by identifying alterations at molecular level associated with physiological trait [10, 11]. Till date, a rich landscape of information about pathogenesis of T2D has been provided [12, 13]. Recent genome-wide association studies (GWAS) in different ethnic population around the world have identified hundreds of T2D susceptible genomic variants, although translating these findings into clinical practice is still challenging [7, 14]. Noteworthy, *CDKAL1*, *HLA-B*, *TCF7L2*, *SLC30A8*, *HHEX*, *IGF2BP2*, *CDKN2A/B*, *EXT2*, and *FTO* genes were found to be associated with T2D in different ethnic populations around the world [15–21]. Very few such studies on T2D are available in Pakistani cohort. Genes like *HHEX/IDE*, *KCNJ11*, *NOTCH2*, *WFS1*, *IRS1*, *CAPN10*, *KCNQ1*, *HNF4A*, *TCF-2/HNF1B*, *IRS-2*, and *TCF7L2* have been studied to be associated with T2D in Pakistani population [22–26].

Pakistani population includes 5 major ethnic groups, namely, Punjabis, Pashtuns, Sindhis, Baluchis, and Muhajirs. Pashtun constitute the major population of Khyber Pakhtunkhwa (KP). They have unique cultural practices and social values, life style, and behaviours that make them a suitable population for such studies. It is hypothesized that genetic mutation spectrum of type 2 diabetes in Pakistani population is different from elsewhere [23, 27]. This study has found deleterious mutations in human leukocyte antigen-B (HLA-B) gene associated with type 2 diabetes in Pashtun ethnic population of Khyber Pakhtunkhwa, Pakistan, using high-throughput sequencing.

The study will help to better understand the pathogenesis of T2D in the study population and to devise modification strategies to overcome/control the burden of this fatal and costly disease.

## 2. Materials and Methods

### 2.1. Participants

**Figure 1:**
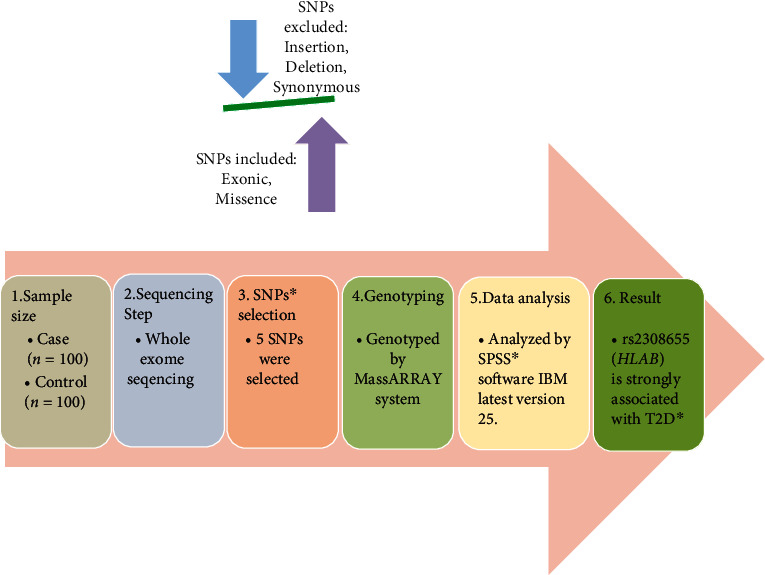
Flow chart of overall study design. SNPs: single-nucleotide polymorphisms; T2D: type 2 diabetes; HLA-B: human leukocyte antigen-B gene.

A total of 200 individuals (diabetic *n* = 100 and nondiabetic *n* = 100) of Pashtun ethnicity belonging from different districts such as Peshawar, Charsadda, Mardan, Bannu, Kohat, Dir, and Swat of Khyber Pakhtunkhwa were included in the study. Patients were registered at Lady Reading Hospital (LRH) Peshawar, Hayatabad Medical Complex (HMC) Peshawar, and Khyber Teaching Hospital (KTH) Peshawar while control samples were collected from specially organized diabetes free medical and screening camps at Rehman Medical Institute (RMI) Hayatabad Peshawar and Diabetic Association Charsadda (DAC). The study period was from July 2018 to July 2019. Inclusion criteria for cases were (i) diabetes diagnosed as per International Diabetes Federation (IDF) etiologic classification, (ii) confirmation that subjects are of Pakistani origin and Pashtun ethnic, and (iii) age above 30 years. Exclusion criteria were (i) mentally ill patients, (ii) age below 30 years, and (iii) diabetes during pregnancy, recent infections, and presence of malignancies. Patient's inclusion and exclusion were according to the previously defined criteria used for Asian populations [28]. Control subjects were healthy individuals from general population with blood sugar level in normal range (<99 mg/dL fasting or 120 mg/dL) [29]. Consent form and thorough demographic, family, and clinical history of all the participants were taken on a carefully designed Proforma. For illiterate participants, who did not understand English, consent form for their understanding was read and explained in local Pashtu language and then signed on his behalf by any of his/her relative/attendant. The study protocols were according to the guidelines of Helsinki declaration (1975), and ethical approval was obtained from the Ethical Committee of the Department of Pharmacy, University of Peshawar. For overall study design, see Figure 1.

### 2.2. Blood Sampling

Three-millilitre whole blood was collected following aseptic procedures from the median cubital vein of study individuals in EDTA tubes (properly labelled) and was stored at -10°C.

### 2.3. DNA Extraction and Pool Preparation

DNA was extracted from 200 *μ*l whole blood samples of T2D patients using WizPrep DNA extraction kit (WizPrep no. W54100). DNA quantification was carried with the help of Qubit™ dsDNA HS Assay kit (Catalog No. Q32851) using Introgen Qubit™ 3, and concentration was adjusted to 10 ng/*μ*L.

### 2.4. Whole Exome Sequencing

Whole exome sequencing (WES) was carried out at the Centre of Genomics, Rehman Medical Institute (RMI), Hayatabad, Peshawar. In order to simplify sequencing process and to reduce cost and time, DNA pools were constructed from 100 diabetic cases and 100 control subjects according to the previously described DNA-pooling protocols [30, 31]. Each pool contains an equimolar amount of DNA (100 ng) from each individual. DNA pools were then subjected to amplification and sequencing via HiSeq2500 platform (Illumina, San Diego, CA, USA) using paired-end libraries (2 × 101 bp).

### 2.5. WES Analysis

A custom-built in-house NGS bioinformatics pipeline was employed in order to move raw sequencing data to final variant calls. Raw FASTQ files produced by the Illumina HiSeq were filtered to separate low quality reads (*Q* > 30) using CASAVA and trimmomatic tool [32, 33]. The filtered reads were then aligned to the reference genome (hg19/GRCh37) using BWA-mem (v 0.7.13) [34, 35]. Base recalibration was carried using GATK (v 3.2.2). Variants were called using GATK Unified Genotyper; the called variants were stored as VCF file. The variants were annotated from variant calling file using ANNOVER [36]. The resulting annotated variant file was loaded in Excel program file for easy understanding, filtering, and analysis of data.

### 2.6. WES Results

**Figure 2:**
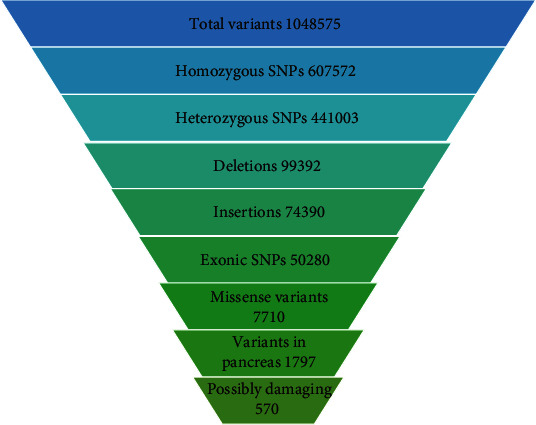
Whole exome sequencing results of study subjects.

Exome sequencing identified a total of 1048575 SNPs including 607572 homozygous SNPs, 441003 heterozygous SNPs, 99392 deletion, 74390 insertion, 50280 exonic SNPs, 7710 missense variants, 1797 variants expressed in pancreas, and 570 possibly pathogenic mutations. Detailed WES results are shown in Figure 2.

### 2.7. Filtration of WES Data

**Figure 3:**
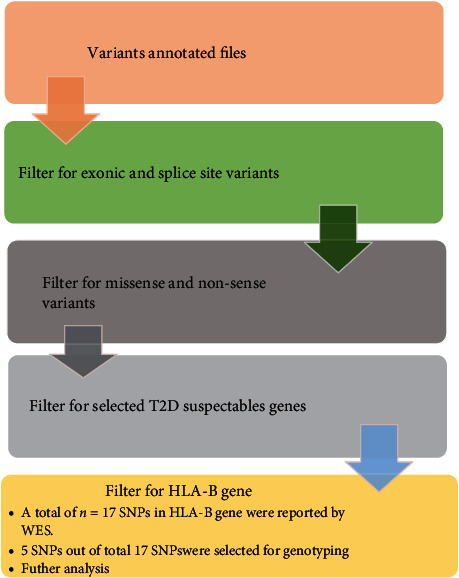
Variant filtration and prioritization pipeline. SNPs: single-nucleotide polymorphisms; T2D: type 2 diabetes; HLA-B: human leukocyte antigen-B gene.

In search of potential T2D, associated variant data was filtered. The flow diagram of data filtration is shown in Figure 3. The annotated Excel files were first manually curated to shortlist exonic and missense variants while synonymous variants were discarded. Resultant file was filtered for T2D susceptible genes (supplementary file 1). HLA-B gene was found to be of interest to be further investigated, among several others. Reported HLA-B variants (*n* = 17) are shown in Table 1.

### 2.8. Validation Trail and Genotyping of HL-B Variants

A total of *n* = 17 SNPs in HLA-B gene were identified using whole exome sequencing (Table 1). In order to validate whole exome sequencing results and to affirm the association of the newly identified HLA-B risk variants with T2D, SNPs were genotyped. Out of *n* = 17 reported SNPs, first 5 SNPs (rs2308655, rs1051488, rs1131500, rs1050341, and rs1131285) were selected for further study. Genotyping of the selected candidate SNPs was carried at the Centre of Genomics, Rehman Medical Institute (RMI), Hayatabad, Peshawar, using Sequenom MassARRAY® system (Agena Bioscience, San Diego, CA) carefully following the manufacturer's guidelines.

### 2.9. Statistical Analysis

Statistical data analysis was performed using IBM SPSS (Statistical Package for Social Sciences version 24). Key variables selected for analysis were age, gender, weight, geographical area, life style, smoking, exercise, occupation, diet, and variants in HLA-B gene. Quantitative variables were checked using independent samples *t*-test. Data for quantitative variables were shown as mean ± standard deviation, while categorical variables of cases and controls were compared using chi-square (*χ*^2^) test. Data for categorical variables were expressed as number and percentage. All reported SNPs were tested for Hardy-Weinberg equilibrium (HWE). Minor allele frequencies (MAFs) between cases and controls were compared using *χ*^2^ test. The association of selected individual SNPs×T2D was checked using binary logistic regression and was also adjusted for combinations within themselves to determine their combined effect. Statistically, a *p* ≤ 0.05 was considered significant.

## 3. Results

### 3.1. Subject Characteristics

Demographic and general characteristics of study subjects are shown in Tables 2 and 3. The prevalence of comorbidities like hypertension, ischemic heart disease, renal failure, retinopathy, and hypercholesterolemia was higher in cases than those of control subjects as shown in Table 2. No significant difference (*t*-test *p* = 0.104) in mean weight of cases and controls (62.64 ± 6.07 vs. 59.55 ± 8.32) was observed. An average normal blood pressure (120/80 mmHg) was observed in patients with T2D; however, an elevated blood pressure (>120/80 mmHg) was observed in patients with comorbidities like ischemic heart disease, renal failure and hypercholestermia. Reference to the patient's general characteristics is shown in Table 3. Sixty-five percent (65%) of patients were males, and 35% were females. The highest incidence of T2D was reported in district Peshawar (53%) followed by district Charsadda (13%), district Mardan (12%), district Swat (08%), district Kohat (06%), district Dir (05%), and district Bannu (03%). Patients included in the study were from different occupations, and most of the patients were doing laborious jobs like driving and farming. Occupation wise, 34% female patients were housewives, 20% were labours, 16% were businessmen, 13% were government servants, 10% were drivers, while 07% were farmers. The highest (34%) prevalence was seen in female patients who were housewives. When patients were asked for family history of T2D, 94% answered “Yes” for family of diabetes while 06% answered “No” for family history of diabetes. Moreover, 85% of patients were nonexercising (sedentary life style), 15% were exercise loving, and none of the patient (0%) was attached with any sport or gym. Majority of (60%) patients were nonsmokers, 21% of patients were using Naswar (a local smokeless tobacco product), while 19% were chain cigarette smokers. The fraction of patients who were taking proper diet in order control diabetes and its complication was 50% while the rest (50%) showed no diet compliance habits.

### 3.2. Minor Allele Frequency (MAF) Analysis

The minor allele frequencies for rs2308655, rs1051488, rs1131500, rs1050341, and rs1131285 were compared between T2D group (*n* = 100) and control group (*n* = 100) using chi-square test. The frequency of the minor allele C was significantly higher in the T2D group as compared to control group (45.0% vs. 13.0%; *p* = 0.006) for rs2308655 in HLA-B gene, while no significant differences in minor allele distribution between cases and control were observed for the other 4 SNPs. For complete detail of MAF comparison between cases and controls, consider Table 4.

### 3.3. Association between SNPs and T2D (SNPs×T2D)

All 5 HLA-B variants (rs2308655, rs1051488, rs1131500, rs1050341, and rs1131285) were checked for T2D association using logistic regression analysis. The frequency of minor C allele was significantly higher in the T2D group as compared to control group (45.0% vs. 13.0%) (*p* = 0.006) for rs2308655 in HLA-B gene. No significant difference in MAF distribution between cases and controls was observed for rs1051488, rs1131500, rs1050341, and rs1131285 (*p* > 0.05). Binary logistic regression analyses showed significant association for SNP rs2308655 (OR = 2.233, CI (95%) = 1.223‐4.077, and *p* = 0.009), while no considerable association was observed for the other 4 SNPs. However, when adjusted for these variants, the association of rs2308655 further strengthened significantly (adjusted OR = 7.485, CI (95%) = 2.353‐23.812, and *p* = 0.001), except for rs1131500, which has no additive effect. For detailed statistical analyses, see Table 5.

## 4. Discussion

The human leukocyte antigen (HLA) gene complex encodes the major histocompatibility complex (MHC) in humans and linked with numerous diseases [37–40] (supplementary file 2). It has three main classes: class I HLAs (A, B, and C), class II HLAs (DQ, DM, DP, DR, and DO), and class III HLAs (CSK2B, SKI2W, C4B, and PBX2) [41]. Mutations in HLA class II alleles are linked with T2D according to previous studies [42–44]. A meta-analysis of 17 genome-wide association studies on T2D performed in African American cohort linked polymorphism in HLA-B (class I gene) with T2D [45]. HLA-B not only increase disease susceptibly but also involve in many adverse drug reaction [46–49]. The present study evaluates the association of genetic variations in HLA-B, a highly polymorphic gene with >3000 variants [50] with T2D in Pashtun ethnic population of Khyber Pakhtunkhwa, Pakistan, using whole exome sequencing.

We performed whole exome sequencing of pooled DNA samples and genotyping of selected SNPs using MassARRAY® system to identify T2D risk variants. Using this approach, our first top notable candidate SNP reported was missense, heterozygous variant rs2308655 (c.1046G>C, p.Cys349Ser) located on short arm of chromosome 6. SIFT and PolyPhen (scores 0.03 and 0.991) predicated rs2308655 variant as deleterious and probably damaging. Second missense, heterozygous, exonic variant reported was rs1051488 (c.985G>A, p.Ala329Thr). SIFT and PolyPhen (scores 0.05 and 0.062) predicated rs2308655 variant as tolerated low confidence and of benign nature. A third missense mutation rs1131500 (c.916G>A, p.Val306Ile) reported was tested nondamaging and benign by SIFT and PolyPhen (scores of 1.00 and 0.001). The last two common missense variants reported were rs1050341 (c.652A>G, p.Ile218Val) and rs1131285 (c.610G>C, p.Glu204Gln); both variants were scored nondamaging and benign by SIFT and PolyPhen.

Our study pinpoints factors like genetic mutations, ethnicity, and environmental and demographic differences involved in the incidence of T2D. The MHC is regarded as highly gene dense and hyperpolymorphic region of human genome [41, 51]. Gene's mutation in MHC region is involved in pathogenesis of diseases like diabetes [52], rheumatoid arthritis [53], cancer [54], multiple sclerosis [55], and psoriasis [56] and also involved in adverse drug reactions [57, 58]. We tested association of genetic polymorphism in HLA-B and T2D susceptibly in Pashtun ethnic population of Khyber Pakhtunkhwa, Pakistan. A large meta-analysis in African American cohort confirms association of T2D and HLA-B polymorphism [45]. Our results suggest strong association of rs2308655 variant in HLA-B gene with T2D. MAF comparison analysis for rs2308655 variant shows that the frequency of minor allele C is much higher in cases than in controls (*p* = 0.006). Regression analysis of rs2308655 also showed significant results (OR = 2.233, CI (95%) = 1.223‐4.077, and *p* = 0.009). In contrast, the other 4 SNPs (rs1051488, rs1131500, rs1050341, and rs1131285) were salient showing no association with T2D. Binary regression analysis results for rs1051488, rs1131500, rs1050341, and rs113128 in HLA-B region were found nonsignificant. However, these genes potentiate the effect of rs2308655, as odd ratios increased significantly when these variants were adjusted with it (adjusted OR = 7.485, CI (95%) = 2.353‐23.812, and *p* = 0.001) (for details, consider Tables 3 and 4).

The exact underlying mechanism of HLA-B polymorphism and incidence of T2D is not yet known, and comprehensive studies on large scale are needed to explore the mechanism rs2308655 variant in T2D.

Furthermore, sociodemographic analysis of cases and controls showed an increase incidence T2D in male compared to female patients. The occurrence of T2D was found high in old age patients compared to younger age patients. Comorbidities like hypertension, ischemic heart disease, renal failure, and hypercholestermia occurrence percentage were found much higher in T2D patients than controls. Most of the patients (94%) were found to have family history of diabetes. Diet control and physical activities (exercise) were recorded poor in patients. Similarly, our data shows that almost 100% patients were belonging from low-income families and doing laborious low-paid jobs.

### 4.1. Study Limitations

Study limitations include limited sample size, and sample collection was restricted only to Khyber Pakhtunkhwa province; our study does not include some intersecting genes that may have strong association with T2D and high percentage of comorbidities in cases. Present study despite of these limitations successfully demonstrated the role of common genetic variants in progression of complex diseases like diabetes in Pakistani cohort. Further, more advance tools of analysis have been used instead of conventional ones.

## 5. Conclusion

The study demonstrated the association of HLA-B gene with T2D in the study population. The findings suggest strong association of SNP rs2308655 with T2D in Pashtun ethnic population. The other HLA alleles, namely, rs1051488, rs1131500, rs1050341, and rs1131285, were shown to have no/very weak (if any) association with T2D. To our knowledge, this study is first of its kind to report T2D risk variants in patients of Khyber Pakhtunkhwa, Pakistan. To overcome this fatal and costly disease, it is recommended that similar projects should be designed on large scale to screen individuals who are genetically susceptible to T2D and awareness campaigns on genetic and environmental risk factors should be initiated in general public. This will help reduce/control the prevalence of the disease.

## Figures and Tables

**Table 1:** HLA-B variants (*n* = 17) reported by whole exome sequencing.

SNP ID	Gene	Variant	Chr	cDNA position	Protein position	HGVSc	HGVSp	SIFT score	PolyPhen score	Alternate allele frequency (%)		Read depth	
										Case	Control	Case	Control
rs2308655	HLA-B	C>C/G	6	1100	349	c.1046G>C	p.Cys349Ser	Del (0.03)	Damaging	87.48	55.22	235	169
rs1051488	HLA-B	C>C/T	6	1039	329	c.985G>A	p.Ala329Thr	Tol (0.05)	Benign	49.07	45	108	80
rs1131500	HLA-B	C>C/T	6	970	306	c.916G>A	p.Val306Ile	Tol (1)	Benign	47.35	49.07	77	54
rs1050341	HLA-B	T>T/C	6	706	218	c.652A>G	p.Ile218Val	Tol (0.51)	Benign	52.16	53.02	301	218
rs1131285	HLA-B	C>C/G	6	664	204	c.610G>C	p.Glu204Gln	Tol (1)	Benign	83.33	85.19	228	257
rs1131275	HLA-B	G>G/C	6	657	201	c.603C>G	p.Asp201Glu	Tol (1)	Benign	85.24	86.05	244	258
rs1050696	HLA-B	A>A/G	6	637	195	c.583T>C	p.Tyr195His	Tol (0.22)	Benign	24.73	29.9	275	291
rs2308466	HLA-B	T>G/A	6 6	614	187	c.560A>C	p.Glu187Ala	Tol (0.57)	Benign	34.67	41.52	349	289
rs2523600	HLA-B	C>T/G		613	187	c.559G>A	p.Glu187Lys	Tol (0.71)	Benign	34.67	41.32	349	288
rs697742	HLA-B	C>C/A	6	593	180	c.539G>T	p.Arg180Leu	Tol (0.66)	Benign	50.99	59.2	402	299
rs9266144	HLA-B	G>G/A	6	592	180	c.538C>T	p.Arg180Trp	Tol (0.2)	Benign	16.34	19.4	410	299
rs151341293	HLA-B	T>T/A	6	581	176	c.527A>T	p.Glu176Val	Tol (0.65)	Benign	25.11	33.04	462	339
rs1050654	HLA-B	G>G/T	6	517	155	c.463C>A	p.Arg155Ser	Tol (0.09)	Benign	37.63	53.15	481	301
rs1140412	HLA-B	G>G/C	6	417	121	c.363C>G	p.Ser121Arg	Tol (0.79)	Benign	61.79	68.91	123	119
rs1071652	HLA-B	C>C/G	6	416	121	c.362G>C	p.Ser121Thr	Tol (0.75)	Benign	61.67	59.02	120	122
rs1050388	HLA-B	C>C/T	6	356	101	c.302G>A	p.Ser101Asn	Tol (1)	Benign	22.08	29.59	240	169
rs1131215	HLA-B	C>C/A	6	346	98	c.292G>T	p.Asp98Tyr	Tol (0.62)	Benign	74.04	72.97	262	185

Abbreviations: SNP: single nucleotide polymorphism; Chr: chromosome; HGVS: human genome association variation; HGVSc: the HGVS coding sequence name; HGVSp: the HGVS protein sequence name; Del: deletion; Tol: tolerated.

**Table 2:** Comorbidities prevalence in study subjects.

Disease	Frequency	
	Cases	Control
Hypertension	34.00%	10.00%
IHD	14.00%	0.00%
Renal failure	5.00%	0.00%
Retinopathy	61.00%	0.00%
Hypercholesterolemia	6.00%	3.01%
HCV	1.00%	0.00%
HBV	0.00%	0.00%

IHD: ischemic heart disease; HCV: hepatitis C virus; HBV: hepatitis B virus.

**Table 3:** Sociodemographic features of cases and controls.

Variables	Case *n* (*f*)	Control *n* (*f*)	*p* value
Gender			0.061
Male	65 (65.0%)	77 (77.0%)	
Female	35 (35.0%)	23 (23.0%)	
Mean age (yrs)	58 ± 12.40	56 ± 13.43	0.951
Mean weight (kg)	62.64 ± 6.07	59.55 ± 8.32	0.104
Occupation			0.112
Labour	20 (20.0%)	14 (14.0%)	
Govt servant	13 (13.0%)	21 (21.0%)	
Business man	16 (16.0%)	18 (18.0%)	
Farmer	07 (7.00%)	16 (16.0%)	
Housewife	34 (34.0%)	23 (23.0%)	
Driver	10 (10.0%)	08 (8.00%)	
Geographical area (district)			0.145
Peshawar	53 (53.0%)	19 (19.0%)	
Charsadda	13 (13.0%)	53 (53.0%)	
Swat	08 (8.00%)	07 (7.00%)	
Dir	05 (4.00%)	05 (5.00%)	
Mardan	12 (12.0%)	10 (10.0%)	
Kohat	06 (6.00%)	03 (3.00%)	
Bannu	03 (3.00%)	03 (3.00%)	
Family history of T2D			0.01
Yes	94 (94.0%)	0.0 (0.00%)	
No	06 (6.00%)	100 (100%)	
Exercise			0.016
Nonexercising	85 (85.0%)	89 (89.0%)	
Walking	14 (14.0%)	04 (4.00%)	
Jogging	01 (1.00%)	05 (5.00%)	
Gym/sport	0.0 (0.0%)	02 (2.00%)	
Smoking			0.178
Cigarette	19 (19.0%)	10 (10.0%)	
Snuff	21 (21.0%)	26 (26.0%)	
Nonsmoking	60 (60.0%)	64 (64.0%)	
Diet control/compliance			0.03
Yes	50 (50.0%)	90 (90.0%)	
No	50 (50.0%)	10 (10.0%)	

kg: kilogram; yrs: years; T2D: type 2 diabetes; *n* (*f*): number (frequency).

**Table 4:** Minor allele frequency comparison between cases and controls.

SNP	Chr(gene)	Minor allele	Minor allele frequency (%)		
			T2D (*n* = 100)	Control (*n* = 100)	*p* value^∗^
rs2308655	6(HLA-B)	C	45.0%	13.0%	0.006
rs1051488	6(HLA-B)	T	49.0%	45.0%	0.396
rs1131500	6(HLA-B)	T	47.0%	49.0%	0.572
rs1050341	6(HLA-B)	T	48.0%	47.0%	0.888
rs1131285	6(HLA-B)	C	17.0%	15.0%	0.7

^∗^Chi-square test; *p* < 0.05 is considered significant.

**Table 5:** Association between selected 5 single-nucleotide polymorphisms and T2D.

SNP	Chr(gene)	Minor allele	OR	CI (95%)	*p* value^∗^
rs2308655	6(HLA-B)	C	2.233	1.233-4.077	0.009
rs1051488	6(HLA-B)	T	1.301	0.745-2.272	0.355
rs1131500	6(HLA-B)	T	1.199	0.687-2.092	0.522
rs1050341	6(HLA-B)	T	0.98	0.562-1.709	0.944
rs1131285	6(HLA-B)	C	1.17	0.537-2.549	0.692
Adjusted odd ratio					
rs2308655	6(HLA-B)	C	7.485	2.353-23.812	0.001
rs1051488	6(HLA-B)	T	0.574	0.00-0.010	0.999
rs1131500	6(HLA-B)	T	1.779	0.00-0.003	1
rs1050341	6(HLA-B)	T	0	0.01-0.002	0.999
rs1131285	6(HLA-B)	C	0.484	0.177-1.322	0.157

^∗^In binary regression analysis: *p* < 0.05 is considered significant; OR: odd ratio; CI (95%): 95% confidence interval; T2D: type 2 diabetes.

## Data Availability

All the needed and necessary information has been provided along with the manuscript. However, the corresponding author can be contacted for any other information related to this paper.

## Supplementary Materials

Supplementary Materials Resultant file was filtered for T2D susceptible genes (supplementary file 1). It has three main classes: class I HLAs (A, B, and C), class II HLAs (DQ, DM, DP, DR, and DO), and class III HLAs (CSK2B, SKI2W, C4B, and PBX2) (supplementary file 2).
