# Supplementary material for: Association of HLA-B Gene Polymorphisms with Type 2 Diabetes in Pashtun Ethnic Population of Khyber Pakhtunkhwa, Pakistan
Source: J Diabetes Res. 2021 Jun 16;2021:6669731. doi: 10.1155/2021/6669731 (PMC8254654; doi:10.1155/2021/6669731)
Supplement: Supplementary Materials — Resultant file was filtered for T2D susceptible genes (supplementary file 1). It has three main classes: class I HLAs (A, B, and C), class II HLAs (DQ, DM, DP, DR, and DO), and class III HLAs (CSK2B, SKI2W, C4B, and PBX2) (supplementary file 2). [file 6669731.f1.zip › 6669731.f1/6669731.f1.pdf]

Gene	Variant	Chr	Coordinate	Type	Genotype	Exonic	Quality	Alt Variant Freq
NOTCH2	T>T/C	1	120572547	snv	het	yes	236.64	25.53
NOTCH2	C>C/T	1	120572572	snv	het	yes	289.64	25.45
NOTCH2	G>G/C	1	120611964	snv	het	yes	5137.64	42.83
GCKR	T>T/C	2	27730940	snv	het	yes	12984.64	75.29
NEUROD1	T>C/C	2	182543455	snv	hom	yes	3021.03	92.78
CAPN10	A>A/G	2	241536126	snv	het	yes	68.64	16.67
CAPN10	T>T/C	2	241538061	snv	het	yes	472.64	20.47
CAPN10	A>G/G	2	241538074	snv	hom	yes	5035.06	98.45
ADCY5	A>A/G	3	123018963	snv	het	yes	1696.64	79.12
WFS1	G>G/A	4	6302519	snv	het	yes	12514.64	77.11
WFS1	G>G/A	4	6302889	snv	het	yes	157.64	14.63
CDKAL1	C>C/T	6	21065449	snv	het	yes	138.64	11.78
HLA-B	C>C/G	6	31322303	snv	het	yes	3397.64	55.22
HLA-B	C>C/T	6	31322911	snv	het	yes	1047.64	49.07
HLA-B	C>C/T	6	31322980	snv	het	yes	816.64	47.35
HLA-B	T>T/C	6	31323337	snv	het	yes	3667.64	52.16
HLA-B	C>C/G	6	31323953	snv	het	yes	7656.64	83.33
HLA-B	G>G/C	6	31323960	snv	het	yes	8381.64	85.24
HLA-B	A>A/G	6	31323980	snv	het	yes	1150.64	24.73
HLA-B	T>A/G	6	31324003	snv	het	yes	6688.1	34.67
HLA-B	C>G/T	6	31324004	snv	het	yes	6695.1	34.67
HLA-B	C>C/A	6	31324024	snv	het	yes	6848.58	50.99
HLA-B	G>G/A	6	31324025	snv	het	yes	485.64	16.34
HLA-B	T>T/A	6	31324036	snv	het	yes	2226.64	25.11
HLA-B	G>G/T	6	31324100	snv	het	yes	5803.64	37.63
HLA-B	G>G/C	6	31324200	snv	het	yes	2900.64	61.79
HLA-B	C>C/G	6	31324201	snv	het	yes	2859.64	61.67
HLA-B	C>C/T	6	31324506	snv	het	yes	1456.64	22.08
HLA-B	C>C/A	6	31324516	snv	het	yes	6461.64	74.04
GCK	G>G/A	7	44185088	snv	het	yes	532.64	21.66
PAX4	T>T/G	7	127251188	snv	het	yes	11347.64	75
PAX4	TA>TA/T	7	127251732	deletion	het	yes	59.6	11.84
SLC30A8	C>C/T	8	118184783	snv	het	yes	1647.64	21.95
GLIS3	G>G/T	9	4118111	snv	het	yes	264.64	78.57
GLIS3	A>G/G	9	4118208	snv	hom	yes	1456.06	100
GPSM1	T>C/C	9	139222174	snv	hom	yes	116.96	100
GRK5	C>C/T	10	121140321	snv	het	yes	111.64	18.05
GRK5	G>G/A	10	121196335	snv	het	yes	231.64	23.21
MOB2	A>G/G	11	1507731	snv	hom	yes	4310.06	93.65
KCNJ11	C>C/T	11	17408630	snv	het	yes	3709.64	48.09
KCNJ11	T>T/C	11	17409572	snv	het	yes	2078.64	53.45
CCND2	C>C/G	12	4388084	snv	het	yes	635.64	32.43
SGCG	T>T/C	13	23898509	snv	het	yes	7512.64	64.5
SGCG	A>A/G	13	23898664	snv	het	yes	13100.64	88.45
TBC1D4	C>T/T	13	75884216	snv	hom	yes	14192.06	95.25
TBC1D4	G>G/A	13	75884290	snv	het	yes	3129.64	37.07

IRS2	C>C/T	13	110435231	snv	het	yes	1033.64	38.62
C2CD4B	A>A/C	15	62456358	snv	het	yes	1114.64	72.31
HNF4A	C>C/T	20	43034693	snv	het	yes	856.64	19.44

Read Depth	Alt Read Depth	Allelic Depths	Transcript
47	12	35,12	NM_024408.3
55	14	41,14	NM_024408.3
558	239	319,239	NM_024408.3
692	521	171,521	NM_001486.3
97	90	7,90	NM_002500.4
36	6	30,6	NM_023083.3
171	35	136,35	NM_023083.3
193	190	3,190	NM_023083.3
91	72	19,72	NM_183357.2
664	512	152,512	NM_006005.3
164	24	140,24	NM_006005.3
518	61	457,61	NM_017774.3
235	136	99,136	NM_005514.6
108	53	55,53	NM_005514.6
77	38	39,38	NM_005514.6
301	154	147,154	NM_005514.6
228	190	38,190	NM_005514.6
244	208	36,208	NM_005514.6
275	68	207,68	NM_005514.6
349	138	90,138,121	NM_005514.6
349	138	90,138,121	NM_005514.6
402	205	197,205	NM_005514.6
410	67	343,67	NM_005514.6
462	116	346,116	NM_005514.6
481	181	300,181	NM_005514.6
123	76	47,76	NM_005514.6
120	74	46,74	NM_005514.6
240	53	187,53	NM_005514.6
262	194	68,194	NM_005514.6
157	34	123,34	NM_021223.2
632	474	158,474	NM_006193.2
228	27	201,27	NM_006193.2
542	119	423,119	NM_173851.2
14	11	3,11	NM_001042413.1
47	47	0,47	NM_001042413.1
5	5	0,5	NR_026964.3
72	13	59,13	NM_005308.2
56	13	43,13	NM_005308.2
189	177	12,177	NM_001172223.1
341	164	177,164	NM_000525.3
174	93	81,93	NM_000525.3
111	36	75,36	NM_001759.3
476	307	169,307	NM_000231.2
563	498	65,498	NM_000231.2
568	541	27,541	NM_014832.2
410	152	258,152	NM_014832.2

145	56	89,56	NM_003749.2
65	47	18,47	NM_001007595.2
391	76	315,76	NM_000457.4

Consequence	cDNA Position	CDS Position	Protein Position
missense_variant	434	137	46
missense_variant	409	112	38
missense_variant	354	57	19
missense_variant,splice_region_variant	1403	1337	446
missense_variant	380	133	45
missense_variant	1706	1510	504
splice_region_variant,intron_variant			0
missense_variant	2192	1996	666
splice_region_variant,intron_variant			0
missense_variant	1167	997	333
missense_variant	1537	1367	456
missense_variant	1393	1226	409
missense_variant,splice_region_variant	1100	1046	349
missense_variant	1039	985	329
missense_variant	970	916	306
missense_variant	706	652	218
missense_variant	664	610	204
missense_variant	657	603	201
missense_variant	637	583	195
missense_variant	614	560	187
missense_variant	613	559	187
missense_variant	593	539	180
missense_variant	592	538	180
missense_variant	581	527	176
missense_variant	517	463	155
missense_variant	417	363	121
missense_variant	416	362	121
missense_variant	356	302	101
missense_variant	346	292	98
upstream_gene_variant			0
missense_variant	1168	962	321
splice_region_variant,intron_variant			0
missense_variant	1203	973	325
missense_variant	2080	1367	456
missense_variant	1983	1270	424
upstream_gene_variant			0
splice_region_variant,intron_variant			0
missense_variant	1144	911	304
missense_variant	218	56	19
missense_variant	1577	1009	337
missense_variant	635	67	23
splice_region_variant,synonymous_variant	875	570	190
splice_region_variant,synonymous_variant	860	705	235
missense_variant	1015	860	287
missense_variant	2802	2455	819
splice_region_variant,intron_variant			0

missense_variant	3684	3170	1057
missense_variant	955	826	276
splice_region_variant,intron_variant			0

Amino Acids	Codons	Transcript HGNC	Canonical	Sift
N/S	aAt/aGt	NOTCH2	YES	tolerated (0.09)
E/K	Gaa/Aaa	NOTCH2	YES	tolerated (0.05)
C/W	tgC/tgG	NOTCH2	YES	tolerated (0.18)
L/P	cTg/cCg	GCKR	YES	tolerated (1)
T/A	Acc/Gcc	NEUROD1	YES	tolerated (0.78)
T/A	Acc/Gcc	CAPN10	YES	tolerated (1)
		CAPN10	YES	
I/V	Atc/Gtc	CAPN10	YES	tolerated (0.25)
		ADCY5	YES	
V/I	Gtc/Atc	WFS1	YES	tolerated (1)
R/H	cGc/cAc	WFS1	YES	deleterious (0.02)
P/L	cCa/cTa	CDKAL1	YES	deleterious (0)
C/S	tGc/tCc	HLA-B	YES	tolerated - low confidence (1)
A/T	Gct/Act	HLA-B	YES	tolerated - low confidence (0.05)
V/I	Gtc/Atc	HLA-B	YES	tolerated - low confidence (1)
I/V	Atc/Gtc	HLA-B	YES	tolerated - low confidence (0.51)
E/Q	Gag/Cag	HLA-B	YES	tolerated - low confidence (1)
D/E	gaC/gaG	HLA-B	YES	tolerated - low confidence (1)
Y/H	Tac/Cac	HLA-B	YES	tolerated - low confidence (0.22)
E/A	gAg/gCg	HLA-B	YES	tolerated - low confidence (0.57)
E/K	Gag/Aag	HLA-B	YES	tolerated - low confidence (0.71)
R/L	cGg/cTg	HLA-B	YES	tolerated - low confidence (0.66)
R/W	Cgg/Tgg	HLA-B	YES	tolerated - low confidence (0.2)
E/V	gAg/gTg	HLA-B	YES	tolerated - low confidence (0.65)
R/S	Cgc/Agc	HLA-B	YES	tolerated - low confidence (0.09)
S/R	agC/agG	HLA-B	YES	tolerated - low confidence (0.79)
S/T	aGc/aCc	HLA-B	YES	tolerated - low confidence (0.75)
S/N	aGc/aAc	HLA-B	YES	tolerated - low confidence (1)
D/Y	Gac/Tac	HLA-B	YES	tolerated - low confidence (0.62)
		MYL7	YES	
H/P	cAc/cCc	PAX4	YES	tolerated - low confidence (0.3)
		PAX4	YES	
R/W	Cgg/Tgg	SLC30A8	YES	tolerated (0.08)
P/Q	cCa/cAa	GLIS3	YES	tolerated (0.91)
S/P	Tcg/Ccg	GLIS3	YES	tolerated (0.49)
		DKFZP434A062	YES	
		GRK5	YES	
R/H	cGt/cAt	GRK5	YES	deleterious (0.03)
L/P	cTg/cCg	MOB2	YES	tolerated - low confidence (1)
V/I	Gtc/Atc	KCNJ11	YES	tolerated (0.11)
K/E	Aag/Gag	KCNJ11	YES	tolerated (0.17)
T	acC/acG	CCND2	YES	
L	ctT/ctC	SGCG	YES	
N/S	aAc/aGc	SGCG	YES	tolerated (1)
V/I	Gta/Ata	TBC1D4	YES	tolerated (0.81)
		TBC1D4	YES	

G/D	gGc/gAc	IRS2	YES	tolerated (0.61)
F/V	Ttc/Gtc	C2CD4B	YES	tolerated (0.18)
		HNF4A	YES	

PolyPhen	ENSP	HGVSc
benign (0.42)	NP_077719.2	NM_024408.3:c.137A>G
benign (0.028)	NP_077719.2	NM_024408.3:c.112G>A
benign (0.013)	NP_077719.2	NM_024408.3:c.57C>G
benign (0)	NP_001477.2	NM_001486.3:c.1337T>C
benign (0)	NP_002491.2	NM_002500.4:c.133A>G
benign (0)	NP_075571.1	NM_023083.3:c.1510A>G
	NP_075571.1	NM_023083.3:c.1990-7T>C
benign (0.001)	NP_075571.1	NM_023083.3:c.1996A>G
	NP_899200.1	NM_183357.2:c.2900+4T>C
benign (0.001)	NP_005996.2	NM_006005.3:c.997G>A
probably damaging (0.995)	NP_005996.2	NM_006005.3:c.1367G>A
benign (0.062)	NP_060244.2	NM_017774.3:c.1226C>T
probably damaging (0.991)	NP_005505.2	NM_005514.6:c.1046G>C
benign (0.062)	NP_005505.2	NM_005514.6:c.985G>A
benign (0.001)	NP_005505.2	NM_005514.6:c.916G>A
benign (0.001)	NP_005505.2	NM_005514.6:c.652A>G
benign (0)	NP_005505.2	NM_005514.6:c.610G>C
benign (0)	NP_005505.2	NM_005514.6:c.603C>G
benign (0.027)	NP_005505.2	NM_005514.6:c.583T>C
benign (0.025)	NP_005505.2	NM_005514.6:c.560A>C
benign (0.024)	NP_005505.2	NM_005514.6:c.559G>A
benign (0.013)	NP_005505.2	NM_005514.6:c.539G>T
benign (0.017)	NP_005505.2	NM_005514.6:c.538C>T
benign (0.027)	NP_005505.2	NM_005514.6:c.527A>T
benign (0.007)	NP_005505.2	NM_005514.6:c.463C>A
benign (0.05)	NP_005505.2	NM_005514.6:c.363C>G
benign (0.001)	NP_005505.2	NM_005514.6:c.362G>C
benign (0.002)	NP_005505.2	NM_005514.6:c.302G>A
benign (0.14)	NP_005505.2	NM_005514.6:c.292G>T
	NP_067046.1	
benign (0)	NP_006184.2	NM_006193.2:c.962A>C
	NP_006184.2	NM_006193.2:c.748-3delT
benign (0.004)	NP_776250.2	NM_173851.2:c.973C>T
benign (0)	NP_001035878.1	NM_001042413.1:c.1367C>A
benign (0)	NP_001035878.1	NM_001042413.1:c.1270T>C
	NP_005299.1	NM_005308.2:c.149-6C>T
benign (0.013)	NP_005299.1	NM_005308.2:c.911G>A
benign (0)	NP_001165694.1	NM_001172223.1:c.56T>C
benign (0.015)	NP_000516.3	NM_000525.3:c.1009G>A
benign (0)	NP_000516.3	NM_000525.3:c.67A>G
	NP_001750.1	NM_001759.3:c.570C>G
	NP_000222.1	NM_000231.2:c.705T>C
benign (0)	NP_000222.1	NM_000231.2:c.860A>G
benign (0.001)	NP_055647.2	NM_014832.2:c.2455G>A
	NP_055647.2	NM_014832.2:c.2384-3C>T

benign (0.025)	NP_003740.2	NM_003749.2:c.3170G>A
benign (0)	NP_001007596.2	NM_001007595.2:c.826T>G
	NP_000448.3	NM_000457.4:c.116-5C>T

HGVSp	dbSNP ID	Conservation Score	Ancestral Allele
NP_077719.2:p.Asn46Ser	rs61788900	2.519	
NP_077719.2:p.Glu38Lys	rs61788901	0.502	
NP_077719.2:p.Cys19Trp	rs11810554	0.738	
NP_001477.2:p.Leu446Pro	rs1260326	0.576	C
NP_002491.2:p.Thr45Ala	rs1801262	0.628	C
NP_075571.1:p.Thr504Ala	rs7607759	-0.875	a
	rs55878652	-0.622	C
NP_075571.1:p.Ile666Val	rs2975766	-1.561	G
	rs4482616	-3.07	G
NP_005996.2:p.Val333Ile	rs1801212	1.481	a
NP_005996.2:p.Arg456His	rs1801208	5.502	g
NP_060244.2:p.Pro409Leu	rs77152992	5.766	C
NP_005505.2:p.Cys349Ser	rs2308655	-0.198	G
NP_005505.2:p.Ala329Thr	rs1051488	0.645	C
NP_005505.2:p.Val306Ile	rs1131500	-0.99	C
NP_005505.2:p.Ile218Val	rs1050341	-1.939	C
NP_005505.2:p.Glu204Gln	rs1131285	0.34	G
NP_005505.2:p.Asp201Glu	rs1131275	0.283	C
NP_005505.2:p.Tyr195His	rs1050696	0.511	
NP_005505.2:p.Glu187Ala	rs2308466	-4.258	g
NP_005505.2:p.Glu187Lys	rs2523600	-1.058	T
NP_005505.2:p.Arg180Leu	rs697742	-1.411	N
NP_005505.2:p.Arg180Trp	rs9266144	-5.021	G
NP_005505.2:p.Glu176Val	rs151341293	-6.462	t
NP_005505.2:p.Arg155Ser	rs1050654	-0.762	
NP_005505.2:p.Ser121Arg	rs1140412	-1.001	
NP_005505.2:p.Ser121Thr	rs1071652	-2.389	
NP_005505.2:p.Ser101Asn	rs1050388	-2.034	
NP_005505.2:p.Asp98Tyr	rs1131215	-2.257	
	rs2908274	0.658	G
NP_006184.2:p.His321Pro	rs712701	-1.012	G
	rs772936097		
NP_776250.2:p.Arg325Trp	rs13266634	-0.325	C
NP_001035878.1:p.Pro456Gln	rs6415788	1.741	T
NP_001035878.1:p.Ser424Pro	rs806052	-1.081	G
	rs28495820	0.032	
	rs2275036	1.139	C
NP_005299.1:p.Arg304His	rs2230349	2.557	G
NP_001165694.1:p.Leu19Pro	rs1881503	1.618	G
NP_000516.3:p.Val337Ile	rs5215	-0.594	T
NP_000516.3:p.Lys23Glu	rs5219	0.716	C
NM_001759.3:c.570C>G(p.=)	rs3217805	-0.352	C
NM_000231.2:c.705T>C(p.=)	rs1800353	-0.065	T
NP_000222.1:p.Asn287Ser	rs1800354	2.747	G
NP_055647.2:p.Val819Ile	rs1062087	-0.161	T
	rs2297203	1.909	G

NP_003740.2:p.Gly1057Asp	rs1805097	0.768	
NP_001007596.2:p.Phe276Val	rs8040712	0.283	C
	rs745975	-2.141	c

Alternate Alleles	Allele Freq	Allele Freq Global Minor	Global Minor Allele	Allele Freq Amr
1	0	0		0
1	0	0		0
1	0	0		0
1	70.67	29.33	T	63.83
2	77.08	22.92	T	74.49
1	11.68	11.68	G	12.54
1	12.04	12.04	C	12.68
2	98.98	1.02	A	98.7
1	80.67	19.33	A	79.39
1	88.44	11.56	G	77.67
1	6.03	6.03	A	7.78
1	8.61	8.6	T	4.9
1	59.22	40.77	C	61.67
1	53.69	46.31	T	49.71
1	54.45	45.55	T	50
1	29.25	29.25	T	28.53
1	81.09	18.91	C	84.01
1	80.23	19.77	G	83.29
1	0	0		0
1	25.88	25.88	G	28.82
1	25.88	25.88	T	28.82
1	49.98	40.54	C	48.13
1	16.69	16.69	A	15.27
1	42.55	42.55	A	45.53
1	0	0		0
1	79.91	18.21	G	76.08
1	14.12	14.12	G	10.66
1	35.64	35.64	T	26.94
1	70.39	29.61	C	75.07
1	41.31	41.31	A	30.84
1	67.05	32.95	T	76.08
1	0	0		0
1	25.52	25.52	T	26.66
1	67.61	32.39	G	76.22
2	99.9	0.1	A	100
2	0	0		0
1	20.51	20.51	T	8.79
1	12.58	12.58	A	9.51
2	94.03	5.97	A	88.33
1	73.06	26.94	C	70.32
1	73.7	26.3	T	70.75
1	21.98	21.98	G	27.81
1	62.98	37.02	T	74.93
1	87.78	12.22	A	93.08
2	71.18	28.81	C	66.43
1	22.6	22.6	A	37.32

1	28.33	28.33	T	41.07
1	73.44	26.56	A	76.22
1	16.87	16.87	T	12.82

Allele Freq Eas	Allele Freq Sas	Allele Freq Af	Allele Freq Eur	EVS Freq All	EVS Coverage
0	0	0	0	0	0
0	0	0	0	0	0
0	0	0	0	0	0
51.88	79.96	90.62	58.95	67.59	6500
91.96	85.28	73.07	61.23	66.94	10700
9.32	21.06	2.87	15.9	12.09	2500
9.32	21.06	4.16	15.9	11.79	2000
100	99.08	99.85	96.92	97.9	2200
87.5	78.43	79.12	78.93	80.09	9200
99.5	85.79	99.39	72.96	79.93	34000
7.94	7.26	3.33	5.27	4.67	7800
15.67	9	7.03	5.76	5	9000
62.6	56.03	68.53	45.03	49.55	5900
55.56	54.19	65.73	38.27	43.4	10100
57.44	55.21	66.03	38.57	43.61	7400
20.34	33.03	39.94	20.97	21.88	8500
81.65	86.71	80.63	73.66	0	0
80.36	86.3	79.58	72.96	46.76	1800
0	0	0	0	0	0
15.57	26.48	28.21	30.52	0	0
15.57	26.48	28.21	30.52	0	0
56.55	57.46	48.94	38.77	0	0
24.01	18.1	11.72	15.51	0	0
38.39	43.66	43.04	42.94	0	0
0	0	0	0	0	0
86.81	85.79	78.06	72.36	59.74	1400
15.97	25.97	7.49	11.83	0	0
27.78	45.81	40.24	33.6	16.84	4700
64.19	82.92	71.63	59.54	48.17	5200
40.28	31.9	72.54	17.69	33.49	2200
35.91	73.62	72.77	78.13	76.89	4800
0	0	0	0	16.26	4200
45.73	25.46	7.41	28.33	22.99	8400
87.4	56.03	60.06	63.02	66.93	1000
100	100	99.62	100	99.87	600
0	0	0	0	0	0
37	19.32	20.57	13.12	14.72	7700
28.27	20.45	0.23	7.55	7.37	6500
99.8	91.1	99.39	87.97	0	0
66.17	60.43	95.46	64.71	73.2	15200
66.17	60.43	97.65	64.71	73.81	6400
8.33	22.6	16.79	37.87	32.63	4100
74.8	77.09	32.53	69.18	57.62	7900
99.9	93.46	70.27	89.46	82.11	6700
79.56	93.35	39.79	85.78	76.5	7900
18.65	20.55	8.09	37.47	31.97	3800

39.28	30.26	9.38	31.61	23.26	500
58.53	68.3	79.35	83.7	0	0
19.15	22.49	8.85	22.46	19.04	14700

EVS Samples	ExAC Freq All	ExAC Freq Afr	ExAC Freq Amr	ExAC Freq Eas	ExAC Freq Sas
0	47.36	32.69	46.64	48.19	49.23
0	46.99	32.29	46.73	47.7	48.94
0	44.93	28.37	43.54	47.15	45.9
6503	64.28	86.46	67.86	50.61	76.05
6503	70.27	73.94	77.24	91.64	85.33
6004	14.74	4.42	7.34	9.81	21.95
6464	19.15	6.54	13.63	12.11	21.93
6468	97.99	99.58	98.62	99.96	98.78
6503	80.09	79.78	79.19	88.06	78.67
6503	78.18	95.55	81.24	99.79	84.33
6503	5.7	3.6	5.81	9.2	6.63
6503	6.55	6.28	4.47	14.86	9.95
6503	52.47	64.99	67.97	62.54	55.94
4220	44.27	59.75	47.76	54.16	53.24
4220	44.72	60.64	48.07	55.86	53.59
6503	23.29	33.83	30.01	21.88	35.52
0	65.4	67.46	78.09	70.3	76.44
6105	57.69	59.81	68.35	61.62	70.37
0	7.01	4.81	8.73	5.85	14.74
0	31.87	30.39	30.39	18.86	37.32
0	31.97	30.33	30.49	18.97	37.32
0	50.26	49.35	53.72	60.43	55.47
0	19.67	15.15	17.22	22.95	20.95
0	41.64	39.95	42.2	39.32	42.8
0	41.32	39.67	45.59	43.71	42.07
5762	65.33	62.63	65.22	85.68	71.17
0	6.98	3.1	7.06	7.46	22.01
6296	22.13	25.9	16.7	20.99	29.43
6297	59.31	61.37	72.25	63.9	73.17
6502	36.8	69.79	44.34	50.08	32.77
6502	75.78	73.22	74.23	42.02	75.54
6257	0	0	0	0	0
6503	28.49	9.12	26.84	43.48	22.9
5571	67.3	63.78	83.59	87.98	57.49
4668	99.97	99.58	100	100	100
0	100	100	100	0	100
6503	15.74	17.22	7.12	38.84	20.73
6503	11.06	2.03	10.18	26.15	19.04
0	90.26	96.81	89.67	99.49	89.29
6493	64.48	91.46	60.97	63.96	62.71
6493	64.71	93.67	61.1	64.01	62.75
6503	32.26	18.8	29.08	8.4	26.14
6503	67.44	39.89	81.82	76.9	71.65
6503	88.17	73.94	93.8	99.92	91.42
5958	82.56	47.98	68.64	81.17	93.28
6052	35.75	13.66	46.56	22.07	24.61

5967	38.2	19.23	57.93	46.68	29.58
0	73.24	83.56	72.3	59.77	69.68
6503	20.08	9.92	9.58	17.55	23.81

ExAC Freq Fin	ExAC Freq Nfe	ExAC Freq Other	ExAC Coverage	ExAC Samples
49.78	48.43	48.06	4900	9016
49.38	48.13	48.11	5100	8477
46.63	46.3	46.45	4400	49819
65.45	58.92	65.78	6800	60623
64.1	62.57	69.18	3800	58380
18.75	16.64	14.78	3800	52662
29.84	21.52	16.67	4100	20298
99.26	97.07	98.28	3900	30829
76.54	79.99	78.44	6200	60385
67.04	71.73	77.42	15100	60700
7.67	5.09	8.06	6900	60625
5.88	5.08	7.27	3600	60675
51.95	45.62	59.09	8400	60548
45.69	37.55	49.67	9100	60181
46.05	37.94	49.55	7400	59516
23.25	17.57	29.36	14700	60440
61.66	59.91	70.97	3300	46065
52.49	52.91	63.19	3500	38735
4.54	5.51	11.74	3400	39520
25.75	33.17	31.22	3800	50647
25.9	33.31	31.63	3800	50746
47.97	46.76	50.41	4400	47243
21.67	19.75	19.87	4200	54180
40.88	41.88	39.9	4800	52805
40.75	40.31	42.4	5500	55216
62.87	61.85	66.31	3600	42096
3.46	4.51	8.66	3100	41804
17.27	21.45	24.79	3200	48625
58.57	52.63	62.23	3600	51245
36.73	27.53	34.07	2600	36274
76.84	80.44	74.67	4200	51282
0	0	0	0	0
37.47	30.33	29.4	9000	60624
69.19	62.92	65.76	2900	47984
100	100	100	2000	42013
0	100	100	41100	22
10.8	13.3	15.56	4700	60296
6.1	9.47	9.51	2900	58159
100	90.88	87.88	6900	6167
52.43	62.6	63.66	11200	60674
52.6	62.61	63.66	10200	60556
35.55	39.22	29.8	3700	60296
67.22	67	66.89	6500	60602
88.15	87.1	87.08	6900	60638
82.51	87.57	83.11	6900	60293
33.4	41.86	36.42	3400	56791

48.67	43.44	36.81	4800	13273
81.25	81.26	77.27	4600	4410
18.61	23	22.03	10700	60594

COSMIC ID

COSM3773408:COSM3773409
COSM1736165
COSM132738
COSM4133785
COSM1179689

COSM3766549:COSM3766550

COSM4408390

COSM4419769

COSM328606

COSM4006327
COSM3734025
COSM4160792
COSM3733865:COSM4593100
COSM3733866:COSM4593368
COSM3761864:COSM4690638:COSM4593366
COSM4419059:COSM4593228
COSM4160793
COSM4006329
COSM4160800
COSM2149151
COSM4160810
COSM1443267
COSM5428531:COSM5428532:COSM5428533

COSM4998633:COSM4430576:COSM4426807:COSM1580374:COSM4426808:COSM4430575:COSM4998632
COSM4162621
COSM3750174:COSM3750175
COSM3763944:COSM3763945
COSM4163535

COSM4987658

COSM3765554
COSM5019156

COSM4147635
COSM3999123
COSM147701

COSM3753571
COSM4128319
COSM5469294:COSM5469295:COSM5469296

COSMIC Allele
C;C
T
C
C
C

C;C

G

A

T

G
C
G
A;G
G;T
A;-;T
A;C
A
T
C
G
T
A
A;A;A

-;-;A;-;A;-;-
T
T;T
G;G
C

A

C
G

G
T
A

T
C
T;T;T

COSMIC Gene
NOTCH2;NOTCH2_ENST00000369342
NOTCH2
NOTCH2
GCKR
NEUROD1

CAPN10_ENST00000391984;CAPN10

ADCY5

WFS1

HLA-B

HLA-B
HLA-B
HLA-B
HLA-B;HLA-B
HLA-B;HLA-B
HLA-B;HLA-B;HLA-B
HLA-B;HLA-B
HLA-B
HLA-B
HLA-B
HLA-B
HLA-B
HLA-B
GCK;GCK_ENST00000403799;GCK_ENST00000395796

PAX4_ENST00000338516;PAX4_ENST00000338516;PAX4;PAX4;PAX4_ENST00000338516;PAX4;PAX4
SLC30A8
GLIS3_ENST00000381971;GLIS3
GLIS3_ENST00000381971;GLIS3
GPSM1_ENST00000392945

GRK5

KCNJ11
CCND2

SGCG
TBC1D4
TBC1D4

IRS2
C2CD4B
HNF4A;Q8N8C9_HUMAN;HNF4A_ENST00000443598

COSMIC Primary Site

haematopoietic_and_lymphoid_tissue;upper_aerodigestive_tract;haematopoietic_and_lymphoid_tissue;pancrea
upper_aerodigestive_tract;haematopoietic_and_lymphoid_tissue;large_intestine;central_nervous_system;thyroi
haematopoietic_and_lymphoid_tissue;urinary_tract;urinary_tract;upper_aerodigestive_tract;haematopoietic_an
pancreas;thyroid
thyroid;prostate

liver;liver

liver

soft_tissue;haematopoietic_and_lymphoid_tissue

prostate;kidney;soft_tissue

urinary_tract;upper_aerodigestive_tract;haematopoietic_and_lymphoid_tissue;thyroid
pancreas;pancreas;thyroid;soft_tissue;large_intestine
upper_aerodigestive_tract;thyroid
pancreas;haematopoietic_and_lymphoid_tissue;thyroid;upper_aerodigestive_tract
pancreas;haematopoietic_and_lymphoid_tissue;thyroid;upper_aerodigestive_tract
upper_aerodigestive_tract;thyroid;soft_tissue;large_intestine;upper_aerodigestive_tract
haematopoietic_and_lymphoid_tissue;upper_aerodigestive_tract;upper_aerodigestive_tract
upper_aerodigestive_tract;thyroid;soft_tissue
urinary_tract;upper_aerodigestive_tract;haematopoietic_and_lymphoid_tissue;thyroid
thyroid
haematopoietic_and_lymphoid_tissue;central_nervous_system
haematopoietic_and_lymphoid_tissue;thyroid;large_intestine
soft_tissue;thyroid;large_intestine
haematopoietic_and_lymphoid_tissue;haematopoietic_and_lymphoid_tissue;haematopoietic_and_lymphoid_tis

pancreas;oesophagus;stomach;oesophagus;central_nervous_system;stomach;oesophagus;oesophagus;pancreas
large_intestine;thyroid;soft_tissue;haematopoietic_and_lymphoid_tissue
thyroid;thyroid
thyroid;thyroid
thyroid

soft_tissue

liver
soft_tissue

thyroid
haematopoietic_and_lymphoid_tissue
stomach;bone;haematopoietic_and_lymphoid_tissue

thyroid
thyroid;upper_aerodigestive_tract
large_intestine;large_intestine;large_intestine

COSMIC Histology

lymphoid_neoplasm;carcinoma;haematopoietic_neoplasm;carcinoma;other;carcinoma;carcinoma;lymphoid_neo

carcinoma;haematopoietic_neoplasm;carcinoma;glioma;other

lymphoid_neoplasm;carcinoma;carcinoma;carcinoma;haematopoietic_neoplasm;glioma;other;haemangioblaston

carcinoma;other

other;adenoma

carcinoma;carcinoma

carcinoma

rhabdomyosarcoma;haematopoietic_neoplasm

carcinoma;carcinoma;haemangioblastoma

carcinoma;carcinoma;haematopoietic_neoplasm;other

pancreatic_intraepithelial_neoplasia_(PanIN);carcinoma;other;rhabdomyosarcoma;carcinoma

carcinoma;other

pancreatic_intraepithelial_neoplasia_(PanIN);haematopoietic_neoplasm;other;carcinoma

pancreatic_intraepithelial_neoplasia_(PanIN);haematopoietic_neoplasm;other;carcinoma

carcinoma;other;haemangioblastoma;carcinoma;carcinoma

haematopoietic_neoplasm;carcinoma;carcinoma

carcinoma;other;haemangioblastoma

carcinoma;carcinoma;haematopoietic_neoplasm;other

other

haematopoietic_neoplasm;glioma

haematopoietic_neoplasm;other;carcinoma

rhabdomyosarcoma;other;carcinoma

haematopoietic_neoplasm;haematopoietic_neoplasm;haematopoietic_neoplasm

carcinoma;carcinoma;carcinoma;carcinoma;glioma;carcinoma;carcinoma;carcinoma;carcinoma

carcinoma;other;rhabdomyosarcoma;haematopoietic_neoplasm

other;other

other;other

other

rhabdomyosarcoma

carcinoma

haemangioblastoma

other

haematopoietic_neoplasm

carcinoma;osteosarcoma;haematopoietic_neoplasm

other
other;carcinoma
carcinoma;carcinoma;carcinoma

ClinVar Accession	ClinVar Alleles
plasm;carcinoma;haematopoietic_neoplasm;carcinoma;other;carcinoma;carcinoma	
na;carcinoma;carcinoma	
RCV000009294.2	C
RCV000038668.7:RCV000155337.1	A:N
RCV000038638.7	A
RCV000117136.2	A
RCV000117896.1	G
RCV000001055.3	T
RCV000117149.2	T
RCV000117148.2	G
RCV000146099.1	T
RCV000009214.3:RCV000009215.3:RCV000020356.1:RCV000146116.2	C
RCV000078407.6	C
RCV000153942.4	G
RCV000118589.2	T
RCV000118588.2	A

RCV000009368.2	T
RCV000117237.2	T

ClinVar Allele Type	ClinVar Significance
germline	
germline	benign
germline	benign
germline	likely benign
germline	benign
germline	
germline	likely benign
germline	likely benign
germline	benign
germline	drug response:benign
germline	benign
germline	benign
germline	likely benign
germline	likely benign

germline	
germline	likely benign

ClinVar Disease Name

Fasting_plasma_glucose_level_quantitative_trait_locus_5

DIABETES_MELLITUS,_TYPE_2,_SUSCEPTIBILITY_TO

Diabetes_mellitus_type_2:Exercise_stress_response,_impaired,_association_with:Permanent_neonatal_diabetes

DIABETES,_TYPE_II,_SUSCEPTIBILITY_TO

ClinVar MedGen	ClinVar OMIM	ClinVar Orphanet	ClinVar GeneReviews
C3150714	613463		
CN169374:CN169374			
CN169374			
CN169374			
CN169374			
CN169374			
CN169374			
CN169374			
C0011860:C3152078:C1833104:CN169374	125853:606176		
CN169374			
CN169374			
CN169374			
CN169374			

CN169374

ClinVar SnoMedCt ID	Regulatory Feature	Google Scholar	PubMed	UCSC Browser
	ENSR00001524148	google	pubmed	ucsc
	ENSR00001524148	google	pubmed	ucsc
	ENSR00001524164	google	pubmed	ucsc
		google	pubmed	ucsc
		google	pubmed	ucsc
		google	pubmed	ucsc
		google	pubmed	ucsc
		google	pubmed	ucsc
		google	pubmed	ucsc
		google	pubmed	ucsc
		google	pubmed	ucsc
	ENSR00001213163	google	pubmed	ucsc
	ENSR00000487828	google	pubmed	ucsc
	ENSR00000487828	google	pubmed	ucsc
	ENSR00000487828	google	pubmed	ucsc
	ENSR00000487828	google	pubmed	ucsc
	ENSR00000487828	google	pubmed	ucsc
	ENSR00000487828	google	pubmed	ucsc
	ENSR00000487828	google	pubmed	ucsc
	ENSR00000487828	google	pubmed	ucsc
	ENSR00000487828	google	pubmed	ucsc
	ENSR00000487828	google	pubmed	ucsc
	ENSR00000487828	google	pubmed	ucsc
	ENSR00000487828	google	pubmed	ucsc
	ENSR00000487828	google	pubmed	ucsc
	ENSR00000487828	google	pubmed	ucsc
	ENSR00000487828	google	pubmed	ucsc
	ENSR00000487828	google	pubmed	ucsc
	ENSR00000487828	google	pubmed	ucsc
	ENSR00001559496	google	pubmed	ucsc
		google	pubmed	ucsc
		google	pubmed	ucsc
		google	pubmed	ucsc
	ENSR00001816392;ENSR00001816393	google	pubmed	ucsc
	ENSR00001816393	google	pubmed	ucsc
	ENSR00001827266	google	pubmed	ucsc
		google	pubmed	ucsc
		google	pubmed	ucsc
	ENSR00001613897	google	pubmed	ucsc
	ENSR00001615863	google	pubmed	ucsc
44054006		google	pubmed	ucsc
		google	pubmed	ucsc
		google	pubmed	ucsc
		google	pubmed	ucsc
		google	pubmed	ucsc
	ENSR00001641968	google	pubmed	ucsc

	google	pubmed	ucsc
ENSR00000409202	google	pubmed	ucsc
	google	pubmed	ucsc
