# Supplementary material for: Association of HLA-B Gene Polymorphisms with Type 2 Diabetes in Pashtun Ethnic Population of Khyber Pakhtunkhwa, Pakistan
Source: J Diabetes Res. 2021 Jun 16;2021:6669731. doi: 10.1155/2021/6669731 (PMC8254654; doi:10.1155/2021/6669731)
Supplement: Supplementary Materials — Resultant file was filtered for T2D susceptible genes (supplementary file 1). It has three main classes: class I HLAs (A, B, and C), class II HLAs (DQ, DM, DP, DR, and DO), and class III HLAs (CSK2B, SKI2W, C4B, and PBX2) (supplementary file 2). [file 6669731.f1.zip › 6669731.f1/6669731.f2.pdf]

HLA loci	HLA allele	Frequency	tagSNP	Allele
	A_01	0.03705	rs74655243	A
	A_0101	0.03583	rs1611703	G
	A_02	0.30250	rs80316590	T
	A_0203	0.02437	rs79172458	C
	A_0206	0.05674	rs117371967	C
	A_0207	0.07335	rs79073194	G
	A_0209	0.00065	rs41540417	A
	A_0217	0.00014	rs41548614	T
	A_03	0.03452	rs112708984	T
	A_0301	0.03214	rs112763429	C
	A_11	0.20630	rs41551619	A
	A_1101	0.18570	rs41547618	A
	A_1102	0.01642	rs41549014	A
	A_23/A_2301	0.00234/0.0023	rs75326938	A
	A_24/A_2402	0.1645/0.1573	rs114398126	T
HLA-A	A_26/A_2601	0.0278/0.0272	rs112946903	T
	A_2602	0.00033	rs80183706	A
	A_29	0.01015	rs41554614	T
	A_2901	0.00950	rs41560216	C
	A_30	0.06820	rs78160437	A
	A_3001	0.06670	rs79293993	A
	A_3002	0.00042	rs1059537	C
	A_31/A_3101	0.03639/0.0363	rs17179220	A
	A_32/A_3201	0.01506/0.01506	rs9258762	A
	A_33/A_3303	0.08242/0.08032	rs76748682	T
	A_3301	0.00211	rs41557315	G
	A_34/A_3401	0.00078/0.00078	rs80145049	A
	A_68	0.00861	rs41562917	T
	A_6801	0.00805	rs41540517	G
	A_6802	0.00033	rs41545116	A
	A_7401	0.00014	rs76828561	A
	B_07	0.03195	rs115289647	T
	B_0702	0.02315	rs2596533	C
	B_0705	0.00763	31452307	C
	B_08/B_0801	0.00875/0.00875	rs2523584	G
	B_13	0.11370	rs71563315	A
	B_1301	0.04257	rs117901686	G
	B_1302	0.07110	rs80317258	T
	B_14	0.00402	rs41555513	A
	B_1502	0.02498	31451122	A
	B_1511	0.02049	31474735	G
	B_1512	0.00136	rs41551517	C
	B_1513	0.00028	31430759	A
	B_1518	0.01637	rs117667673	T

HLA-B	B_1519	0.00019	rs41545517	A
	B_1527	0.00893	rs41562013	A
	B_18	0.00388	rs9266175	T
	B_1801	0.00360	rs9266183	C
	B_27	0.02334	rs76334813	T
	B_2704	0.01048	rs41553413	C
	B_2705	0.00908	rs41558317	G
	B_3502	0.00239	rs41558819	A
	B_37/B_3701	0.01413/0.01413	rs3819276	T
	B_3801	0.00535	rs147748716	T
	B_39/B_3901	0.02030/0.01735	rs41552422	A
	B_4001	0.09594	rs1131168	C
	B_41	0.00103	rs41544517	T
	B_44	0.03784	rs9266195	T
	B_4402	0.00861	rs112812799	C
	B_4403	0.02821	rs113858764	T
	B_45/B_4501	0.001403/0.00140	rs16899066	C
	B_46/B_4601	0.07882/0.07882	rs41546313	C
	B_47/B_4701	0.000327/0.00032	rs41553914	A
	B_48/B_4801	0.03293/0.02966	rs2308488	T
	B_49/B_4901	0.00168/0.00168	rs77855453	T
	B_50/B_5001	0.00650/0.00650	rs41552112	A
	B_51	0.07045	rs1050502	A
	B_5142	0.00173	rs41545013	C
	B_54/B_5401	0.03106/0.03106	rs41547515	C
	B_57/B_5701	0.01338/0.01338	rs41557415	G
	B_58/B_5801	0.04902/0.04902	rs75412754	C
	B_81/B_8101	0.00042/0.00042	rs41556216	A
	C_01	0.15120	rs41557015	A
	C_0102	0.14100	rs2074494	T
	C_0106	0.00098	rs112170844	A
	C_0108	0.00023	rs41551019	A
	C_02/C_0202	0.00865/0.0086	rs41562921	T
	C_03	0.22070	rs34592426	C
	C_0302	0.05188	rs74703819	T
	C_0303	0.07901	rs1131122	T
	C_0304	0.08897	rs35840219	C
	C_04	0.06708	rs41561715	T
	C_0401	0.06016	rs41547312	T
	C_0403	0.00575	31351531	A
	C_05/C_0501	0.00818/0.00790	rs9264769	A
	C_06/C_0602	0.1052/0.1052	rs4406273	A
	C_07	0.17030	rs9264608	A
	C_0701	0.00561	rs77326983	C
	C_0702	0.14460	rs76203972	C

HLA-C	C_0704	0.00978	rs77138865	A
	C_0706	0.00842	rs41542414	T
	C_0766	0.00117	rs74500356	T
	C_0767	0.00014	rs75032224	A
	C_08	0.10830	rs11547346	G
	C_0802	0.00370	rs41544520	T
	C_0803	0.00917	rs111613560	T
	C_0822	0.01272	rs76294545	T
	C_1202	0.03639	rs111254458	A
	C_1203	0.01637	rs41553316	A
	C_14	0.05754	rs72558161	C
	C_1402	0.04303	rs76235944	T
	C_1403	0.01431	rs117113479	G
	C_15	0.04626	rs41561913	A
	C_1502	0.03733	rs1065406	T
	C_1505	0.00781	rs41543119	A
	C_1513	0.00019	rs41547622	G
	C_16	0.00257	rs28367580	G
	C_17/C_1701	0.00103/0.00103	rs41544314	T
HLA-DRB1	DRB1_01	0.02390	rs17885011	T
	DRB1_0101	0.02175	rs112460460	A
	DRB1_03	0.04584	rs9269941	T
	DRB1_0301	0.04289	rs67476479	C
	DRB1_0317	0.00253	rs17884787	C
	DRB1_04	0.11080	rs17879020	A
	DRB1_0405	0.04411	rs117433623	G
	DRB1_0406	0.02517	32365630	C
	DRB1_07/DRB1_0701	0.09150/0.0915	rs17885382	T
	DRB1_08	0.07662	rs78817721	A
	DRB1_0803	0.06900	32551980	C
	DRB1_09/DRB1_0901	0.15325/0.15325	rs17884840	A
	DRB1_10/DRB1_1001	0.01492/0.01492	rs3205588	T
	DRB1_11/DRB1_1101	0.06465/0.05726	rs1059584	T
	DRB1_12	0.11290	rs17884840	T
	DRB1_1210	0.00309	rs17878475	T
	DRB1_13	0.05544	rs113096319	T
	DRB1_1301	0.01497	rs115427852	G
	DRB1_1302	0.03541	rs116103474	A
	DRB1_1312	0.00449	rs35067512	G
	DRB1_14	0.06563	rs1059615	C
	DRB1_1405	0.02250	rs17879981	T
	DRB1_15	0.15756	rs9270299	A
	DRB1_1501	0.12740	rs113397833	A

	DRB1_1502	0.02910	rs116980366	G
	DRB1_16	0.02699	rs116602178	G
	DRB1_1602	0.02615	rs116502595	T
	DQB1_02	0.12500	rs1130370	T
	DQB1_0201	0.04168	rs1794280	T
	DQB1_0202	0.08331	rs2647032	C
	DQB1_03	0.42470	rs1130380	G
	DQB1_0301	0.20600	rs1049058	A
	DQB1_0302	0.05552	rs113588380	T
	DQB1_0303	0.16160	rs114662434	T
	DQB1_04	0.04760	rs1130391	T
HLA-DQB1	DQB1_0401	0.03345	rs41540813	A
	DQB1_05	0.14340	rs79326791	G
	DQB1_0501	0.04542	rs1049074	T
	DQB1_0502	0.05510	rs76051596	G
	DQB1_0503	0.04285	rs1794281	G
	DQB1_06	0.25930	rs1049062	G
	DQB1_0601	0.09379	rs3204379	T
	DQB1_0602	0.10690	rs1049062	A
	DQB1_0603	0.01703	rs113791489	T
	DQB1_0604	0.01852	rs17206126	C
	DQA1_01	0.57670	rs80318326	T
	DQA1_0101	0.05370	rs4476870	G
	DQA1_0102	0.27860	rs80176586	A
	DQA1_0103	0.13400	rs36219699	A
	DQA1_0104	0.08687	rs35087390	A
	DQA1_02/DQA1_0201	0.07277/0.07277	rs1142326	A
	DQA1_03	0.18470	rs12722074	A
HLA-DQA1	DQA1_0302	0.10170	rs12722040	C
	DQA1_04/DQA1_0401	0.00523/0.00523	rs1142338	T
	DQA1_05	0.10760	rs9279910	G
	DQA1_0505	0.06184	rs1047992	A
	DQA1_0506	0.00290	rs41555012	G
	DQA1_0508	0.00547	rs41544114	T
	DQA1_0509	0.00042	rs41545012	A
	DQA1_06/DQA1_0601	0.0530/0.0530	rs1048134	G
	DPB1_02	0.25350	rs2295119	T
	DPB1_0202	0.05609	rs118044406	G
	DPB1_0401	0.10600	rs2068205	T
	DPB1_0402	0.05019	rs76893992	A
	DPB1_05/DPB1_0501	0.3686/0.3686	rs80315764	T
HLA-DPB1	DPB1_09/DPB1_0901	0.01431/0.01431	rs115910061	T
	DPB1_13/DPB1_1301	0.04519/0.03527	rs112248439	A
	DPB1_15/DPB1_1501	0.00182/0.00182	rs41559424	T

	DPB1_17/DPB1_1701	0.04603/0.04603	rs16868789	G
	DPB1_19/DPB1_1901	0.00763/0.00763	rs114283055	A
	DPB1_21/DPB1_2101	0.01137/0.01137	rs117239359	T
	DPB1_26/DPB1_2601	0.0022/0.0022	rs41545021	A
	MICA_002:01	0.1388	rs79366716	C
	MICA_004	0.03401	rs9295989	C
	MICA_008:01	0.14	rs3128981	G
	MICA_008:04	0.1154	rs77577410	G
	MICA_009:02	0.006783	rs6912525	C
MICA	MICA_010:01	0.1828	rs2263323	T
	MICA_012:01	0.05983	rs114905838	C
	MICA_027	0.08228	rs115368838	C
	MICA_045	0.0508	rs115827739	G
	MICA_049	0.03822	rs80218014	C
	MICA_071	0.01305	31490474	A
	DPA1_01/DPA1_0103	0.3939/0.3939	rs1042434	G
	DPA1_02	0.59890	rs2308911	G
HLA-DPA1	DPA1_0201	0.11350	rs1042177	T
	DPA1_0202	0.48540	rs41552628	T
	DPA1_03/DPA1_0301	0.00112/0.00112	rs2308917	G
	DPA1_04/DPA1_0401	0.00608/0.00608	rs41548220	A
	MICB_003	0.03752	rs41293887	A
	MICB_004:01	0.09131	rs3132465	G
MICB	MICB_005:03	0.04949	rs76329749	A
	MICB_005:06	0.00711	rs115339407	A
	MICB_008	0.0557	rs3134900	G
	MICB_014	0.0356	rs41273040	A
	DMB_01:01	0.646	rs1042337	A
HLA-DMB	DMB_01:03	0.332	rs1042337	G
	DMB_01:07	0.01736	rs6920593	A
	DOA_01:01:01	0.3545	rs378352	A
HLA-DOA	DOA_01:01:02	0.4456	rs365066	C
	DOA_01:01:04	0.1828	rs6913008	T
	DOA_01:02	0.008373	33087093	C
	DOB_01:01:01	0.05714	rs7383287	G
	DOB_01:02	0.07208	rs9784869	T
HLA-DOB	DOB_01:03	0.005145	rs2857105	C
	DOB_01:04	0.0762	rs2070120	A
	DOB_01:05	0.0203	rs28724895	C
	DRA_01:01:01	0.7172	rs8084	C
HLA-DRA	DRA_01:01:02	0.01681	rs11544315	T
	DRA_01:02:02	0.2494	rs7192	T
	DRA_01:02:03	0.01656	rs3135391	A
HLA-E	E_01:01:01	0.4023	rs1264457	A
	E_01:03:02	0.3076	rs1059510	T
	F_01:01:01	0.4367	rs113770962	G

HLA-F	F_01:01:02	0.1985	rs2076183	A
	F_01:01:03	0.3245	rs1736922	T
	F_01:03:01	0.03977	rs1736924	C
HLA-G	G_01:01:01	0.403	rs1130363	A
	G_01:01:03	0.1274	rs3873252	T
	G_01:01:12	0.01423	rs9258495	T
	G_01:03:01	0.0109	rs41551813	T
	G_01:04:01	0.2228	rs12722477	A
	G_01:05N	0.0635	rs56314023	A
	G_01:06	0.03634	rs12722482	T
HLA-H	H_01:01	0.4909	rs113058541	A
	H_01:02	0.05936	rs7768484	T
	H_02:01	0.04818	rs116722566	C
	H_02:02	0.01478	rs2524026	A
	H_02:03	0.01595	rs9258788	T
	H_02:04	0.1987	rs9259290	T
	H_02:05	0.109	rs112003535	T
HLA-K	K_01:01:01	0.5452	rs438610	C
	K_01:02	0.4132	rs112201735	C
	K_01:03	0.01247	rs114214384	A
HLA-L	L_01:01:01	0.5586	rs113471469	G
	L_01:01:02	0.06183	rs3129697	G
	L_01:02	0.3796	rs111858555	G
HLA-P	P_01:01	0.1724	rs112697085	T
	P_02:01	0.8147	rs1077433	G
TAP1	TAP1_01:01	0.7561	rs12527715	T
	TAP1_02:01	0.1644	rs4711312	G
TAP2	TAP2_01:03	0.02468	rs4148876	A
	TAP2_01:04	0.1741	rs104893997	T
	TAP2_02:01:01	0.01439	rs1042116	A
	TAP2_02:01:02	0.2889	rs241441	C

**Frequency**	**Rsquare**	**Equivalent(r2>=0.**	**Disease related with type**
0.03688	1	115	
0.03621	0.987	115	
0.30330	1.000	129	primary nodal generalized osteoarthritis ovarian cancer chronic hepatitis
0.02409	0.924	1	
0.06282	0.834	3	
0.07372	1.000	8	
0.00066	1.000	1	
0.00014	1.000	1	
0.03471	1.000	149	
0.03467	0.931	73	
0.20480	1.000	66	
0.20480	0.894	16	
0.01651	1.000	2	
0.00236	1.000	1	
0.16380	0.994	179	
0.02844	0.973	100	
0.00033	1.000	2	
0.01023	1.000	119	
0.00958	1.000	123	retinopathy
0.06712	1.000	165	
0.06594	0.994	179	
0.00042	1.000	2	
0.03691	0.972	14	carbamazepine
0.01502	0.997	133	
0.08014	0.980	70	
0.00212	1.000	2	
0.00080	1.000	3	
0.00840	1.000	16	
0.00807	1.000	17	
0.00033	1.000	1	
0.00014	1.000	1	
0.03391	0.900	9	alzheimer's disease,osteoporosis
0.02537	0.864	3	
0.00819	0.921	38	
0.00875	1.000	75	Sjogren's syndrome,early onset myasthenia gravis
0.11390	1.000	18	
0.04332	0.962	131	
0.07003	0.971	75	
0.00403	1.000	5	
0.02503	0.957	18	epilepsy,lamotrigine induced sjs/ten
0.02205	0.895	3	
0.00145	0.871	2	
0.00028	1.000	1	
0.01834	0.857	2	

0.00019	1.000	1	
0.00928	0.964	1	
0.00385	1.000	4	
0.00385	0.926	4	
0.02068	0.866	3	Acute anterior uveitis,ankylosing spondylitis
0.01139	0.917	4	
0.00952	0.950	32	
0.00239	1.000	1	hypertension
0.01416	1.000	29	
0.00560	0.918	3	
0.01819	0.957	1	
0.09616	1.000	49	
0.00103	1.000	1	
0.03760	1.000	6	
0.00903	0.932	13	
0.03205	0.856	8	
0.00141	1.000	1	
0.07877	1.000	6	
0.00033	1.000	4	
0.03188	0.945	34	
0.00169	1.000	1	
0.00642	1.000	14	
0.07052	1.000	9	Behçet's disease,papillary thyroid carcinoma,cleft lip
0.00174	1.000	1	
0.03113	1.000	6	
0.01341	1.000	48	Abacavir hypersensitivity,flucloxacillin
0.05202	0.923	68	allopurinol
0.00042	1.000	1	
0.14970	1.000	52	
0.14960	0.932	21	
0.00104	0.954	1	
0.00019	1.000	1	
0.00872	1.000	70	
0.22120	1.000	27	
0.05206	0.979	100	
0.07917	1.000	3	
0.08977	0.960	1	
0.06635	0.999	27	
0.06041	1.000	23	
0.00571	0.976	33	
0.00849	0.945	10	
0.10490	0.994	48	psoriasis
0.17120	1.000	156	
0.00505	0.854	2	age-related macular degeneration
0.14680	0.989	182	

0.00985	1.000	73	
0.00848	1.000	93	
0.00118	1.000	1	
0.00014	1.000	1	
0.10510	0.961	109	
0.00382	0.975	2	
0.00928	0.995	1	
0.01282	1.000	1	
0.03666	0.999	3	
0.01645	1.000	5	
0.05749	1.000	41	
0.04289	0.985	11	
0.01431	1.000	95	
0.04557	1.000	18	
0.03728	0.995	9	
0.00787	1.000	39	
0.00019	1.000	1	
0.00259	1.000	8	
0.00104	1.000	35	
0.02414	1.000	78	
0.02133	0.971	65	coronary heart disease, dupuytren's disease, dermatomyositis
0.04559	1.000	20	
0.04289	0.993	23	autoimmune hepatitis, lofgren's, polymyositis, encephalomyelitis
0.00255	1.000	2	
0.11170	1.000	16	end-stage renal disease , hashimoto's
0.04257	0.805	1	
0.02320	0.823	1	
0.09231	1.000	27	rheumatic heart disease, chlorpromazine-
0.08078	0.940	2	
0.06053	0.863	73	
0.15460	1.000	14	gelatin allergy , antineutrophil cytoplasmic antibody-associated vasculitis
0.01507	1.000	39	
0.06430	1.000	1	
0.11370	1.000	5	
0.00307	1.000	1	
0.04991	0.858	2	
0.01450	0.938	17	
0.03546	0.940	10	
0.00444	0.970	1	
0.05522	0.903	1	
0.02249	1.000	1	
0.15910	1.000	11	bronchopulmonary aspergillosis,keloid, leprosy, late onset myasthenia gravis, multiple sclerosis, goodpastures syndrome , allergic bronchopulmonary aspergillosis,glaucoma
0.13210	0.842	3	

0.02587	0.853	5	
0.02615	0.957	16	
0.02624	0.932	11	melioidosis
0.12500	1.000	15	chronic hepatitis C, dermatitis herpetiformis, iga deficiency, irritable bowel syndrome
0.04351	0.920	22	
0.08337	1.000	7	
0.42450	1.000	6	alopecia areata, creutzfeldt-jakob disease, pollen allergy,latex allergy
0.20640	0.998	4	
0.05772	0.952	48	
0.16330	0.925	4	
0.04765	1.000	28	
0.03347	1.000	2	
0.14350	1.000	8	
0.04545	1.000	56	
0.05514	1.000	4	moyamoya disease
0.04130	0.928	15	
0.30220	0.812	1	
0.09386	1.000	8	
0.10710	0.999	3	narcolepsy, gastric cancer
0.01511	0.910	7	peanut allergy
0.01871	0.980	30	nonalcoholic fatty liver disease
0.57670	1.000	23	
0.04415	0.857	1	
0.27860	1.000	1	abdominal aortic aneurysm
0.13400	1.000	5	
0.08687	1.000	5	
0.07277	1.000	3	breast cancer
0.18460	1.000	7	lung adenocarcinoma,pollen-induced allergic rhinitis
0.10170	1.000	1	
0.00524	1.000	1	
0.10760	1.000	9	
0.06774	0.916	2	
0.00290	1.000	1	
0.00547	1.000	1	
0.00042	1.000	1	
0.05824	0.913	3	
0.25460	0.946	2	
0.05375	0.949	2	
0.09987	0.872	5	
0.05189	1.000	10	
0.36170	0.964	46	
0.01473	0.958	1	hepatitis C virus-associated hypertrophic cardiomyopathy
0.04253	0.858	1	
0.00189	1.000	5	

0.04629	0.988	38	
0.00791	0.964	39	
0.01071	0.956	7	
0.00229	1.000	1	
0.1394	0.9602	105	
0.03394	0.85781	8	
0.111	0.78	2	
0.1348	0.83996	1	
0.008005	0.85144	24	
0.1871	0.96619	12	
0.05942	0.99212	76	
0.07208	0.84669	7	
0.05556	0.88431	136	
0.03878	0.91713	6	
0.01409	0.91048	43	
0.39390	1.000	88	
0.59890	1.000	78	
0.11350	1.000	7	asthma
0.48540	1.000	25	
0.00112	1.000	1	
0.00608	1.000	9	
0.04015	0.9332	9	
0.09245	0.9801	21	
0.05492	0.88386	3	
0.007578	0.92578	16	
0.0557	1	21	
0.0379	0.89424	1	
0.651	0.92595	2	
0.331	0.99501	2	
0.01736	1	74	
0.3545	1	9	
0.4525	0.97309	5	
0.1744	0.82813	4	
0.008888	0.89878	2	
0.05714	1	1	
0.07758	0.90903	5	
0.005847	0.88073	10	
0.07695	0.98125	4	
0.02035	0.99295	9	
0.7172	1	18	
0.01681	1	35	
0.2659	0.91596	14	
0.01656	1	7	
0.4023	1	1	
0.3076	1	5	
0.4037	0.86346	8	

0.1985	1	42
0.325	0.99772	36
0.03977	1	97
0.4203	0.93402	63
0.1281	0.99422	206
0.01423	1	98
0.0109	1	32
0.2405	0.90807	42
0.07294	0.86215	69
0.03634	1	102
0.4909	1	8
0.05936	1	6
0.04281	0.892333	92
0.01271	0.872066	27
0.01558	0.97697	124
0.1759	0.815132	5
0.09393	0.860421	11
0.5452	1	28
0.4132	1	31
0.01247	1	96
0.5561	0.97858	32
0.06183	1	84
0.3796	1	50
0.1638	0.94073	124
0.8247	0.93571	143
0.782	0.81047	2
0.1773	0.85807	4
0.02468	1	2
0.1741	1	1
0.01681	0.86376	1
0.3033	0.91827	4

Pubmed ID

21422676 , 22258792 , 18418397

16998491

21428769

17176470 , 17498269

16998491, 22590570

20831525 , 20235790

22879410,23749180

16948645

25062414, 24308748, 10090614

18184080, 19483685
24732692

25087609

18515573

20842443,22991974,22886382

20933106 , 18513341 , 14511932 , 25339670

24603486 , 19254248 , 9698788 , 18256425

23886662 , 12197571,17578052,9451597,3496181

11312025 , 25030430

11031343,18976433,20003324,22590574,24278027,18502098
, 17561243,16005081

9757914

18028350 , 18028350,22291608,21490506

21692766,3532932,9641569,12209103

9409445

11179016 , 15691311
23443026
25156535

17182961

21245432
20061363, 14990915

18186799

21814517
